# Vaccination decision-making of immigrant parents in the Netherlands; a focus group study

**DOI:** 10.1186/s12889-015-2572-x

**Published:** 2015-12-10

**Authors:** Irene A. Harmsen, Helien Bos, Robert A. C. Ruiter, Theo G. W. Paulussen, Gerjo Kok, Hester E. de Melker, Liesbeth Mollema

**Affiliations:** National Institute for Public Health and the Environment (RIVM), Centre for Infectious Disease Control, P.O. Box 1 3720, BA Bilthoven, The Netherlands; Department of Work & Social Psychology, Maastricht University, Universiteitssingel 40, 6200 MD Maastricht, The Netherlands; Department of Earth and Life Sciences, VU University of Amsterdam, De Boelelaan 1085-1087, 1081 HV Amsterdam, The Netherlands; TNO (Netherlands Organization for Applied Scientific Research), Healthy Living, Wassenaarseweg 56, NL-2333 AL Leiden, The Netherlands

**Keywords:** Childhood vaccination, Decision-making, Non-Western, Ethnic background

## Abstract

**Background:**

Although the vaccination coverage in most high income countries is high, variations in coverage rates on the national level among different ethnic backgrounds are reported. A qualitative study was performed to explore factors that influence decision-making among parents with different ethnic backgrounds in the Netherlands.

**Methods:**

Six focus groups were conducted with 33 mothers of Moroccan, Turkish and other ethnic backgrounds with at least one child aged 0–4 years. Data were analysed using thematic analysis.

**Results:**

Parents had a positive attitude towards childhood vaccination and a high confidence in the advices of Child Vaccine Providers (CVPs). Vaccinating their children was perceived as self-evident and important. Parents do perceive a language barrier in understanding the provided NIP-information, and they had a need for more NIP- information, particularly about the targeted diseases. Another barrier parents perceived was the distance to the Child Welfare Center (CWC), especially when the weather was bad and when they had no access to a car.

**Conclusion:**

More information about targeted diseases and complete information regarding benefits and drawbacks of the NIP should be provided to the parents. To fulfill parents’ information needs, NIP information meetings can be organized at CWCs in different languages. Providing NIP information material in Turkish, Arabic and Berber language with easy access is also recommended. Providing information tailored to these parents’ needs is important to sustain high vaccination participation, and to ensure acceptance of future vaccinations.

## Background

Throughout the world, childhood immunization is a major public health intervention for preventing disease and mortality [1]. In the Netherlands, the overall vaccination coverage among newborns is 95 %. The vaccination coverage among young-adolescent girls (they get vaccinated against cervical cancer caused by human papillomavirus (HPV), is 59 % [2]. The National Immunization Programme (NIP) is coordinated by the National Institute for Public Health and the Environment (RIVM). The NIP targets 12 diseases (i.e., polio, diphtheria, tetanus, pertussis, rubella, measles, mumps, disease caused by *Haemophilus influenzae* type b, meningococcal C disease, hepatitis B, pneumococcal disease and cervical cancer caused by HPV), is non-mandatory, and free of charge [2]. About 90 % of all children in the Netherlands periodically visit the local Child Welfare Centers (CWCs) for free health check-ups, and that is also where they receive the NIP vaccinations Most parents are positive towards the NIP. Groups with lower vaccination coverage rates in the Netherlands are people refusing vaccinations based on anthroposophical beliefs [3], religion [4], or other beliefs like the possible side effects of vaccines [5]. Mixed findings were reported about parents with different ethnic backgrounds. Mollema et al. [6] and Van Lier et al. [7] showed that the participation in childhood vaccination is somewhat lower for certain ethnicities (ranging from 1.2 % lower for both parents born in Morocco to 3.5 % lower for both parents born in other non-Western countries), a pattern also found among minority groups in other countries [8, 9]. Luman et al. [10] and Chu et al. [11] indicated that ethnic minority groups sometimes have limited access to primary care and have misconceptions about risks and benefits of vaccination. Language barriers and a more frequent change of residence and therefore not receiving (follow up) invitations for vaccination, were also suggested as possible explanations for slightly lower full coverage rates [6, 7].

In addition, vaccinations given abroad are not registered in the Netherlands, which might result in under-reporting of the actual vaccination coverage among different ethnicities living in the Netherlands [7]. Besides, Streefland et al. [1] indicated that parents with different ethnic backgrounds perceive vaccination as self-evident, and thereby suggests that compliance rates in this group might even be higher than those of native Dutch parents.

So far, no qualitative study explored factors that influence vaccination decision-making among parents with different ethnic backgrounds in the Netherlands. Therefore, this qualitative focus group study was conducted, particularly among parents with Moroccan and Turkish nationality, the two largest non-Dutch ethnic groups in the Netherlands [12]. We also wanted to get more insight into CWC-visitation, perception of the current provided NIP-information, information need of the parents, and attitude towards possible future vaccinations within the NIP. Better understanding of factors influencing vaccination decision-making of these parents is needed to gain more insight into how vaccine uptake in these groups can best be promoted.

## Methods

### Study participants

Six focus groups were conducted with mothers of different ethnic backgrounds who had at least one child aged 0–4 years old. The total number of participants was 33, and all participants were female. Two groups (*N* = 7 and 7) consisted of mothers of Moroccan nationality, two groups (*N* = 4 and 3) of Turkish mothers and two groups (*N* = 6 and 6) comprised mothers of different nationalities (Netherlands (*n* = 6), Morocco (*n* = 2), Afghanistan (*n* = 1), Somalia (*n* = 1), Poland (*n* = 1), Belgium (*n* = 1)). Moroccan and Turkish mothers participated in separate focus groups to create transparency and avoid obstacles due to cultural differences. The two mixed groups were used to study vaccination decision-making among persons with other ethnic backgrounds than Moroccan and Turkish. At the time of the focus groups, all mothers had lived in the Netherlands for at least 1 year.

### Study setting

All six focus groups were held during regular mother-baby group meetings organized by the welfare organization ‘Cumulus Welzijn’ in Utrecht, the Netherlands. ‘Cumulus Welzijn’ provides activities, services, and facilities to local residents, including parental support group meetings where the development of the new-born baby is stimulated [13]. All mothers who were present at the respective regular mother-baby meeting participated in the focus groups. A total of six focus groups were considered to be sufficient because in the final two focus groups no new information was generated and data saturation was reached. The focus groups were conducted in April and May 2012, and each focus group discussion lasted one hour.

### Procedure

All focus groups were facilitated by a moderator and an assistant. Besides the moderator, the assistant, and the participants, a female group leader (who normally leads the mother-baby group meetings at ‘Cumulus Welzijn’) was present. In the Moroccan and Turkish groups, this group leader translated the conversation for mothers who had difficulties with the Dutch language. She had no role in leading the discussion. Informed consent was obtained and focus group participants were offered a gift voucher of €10 as a gratitude for their participation. Confidentiality of participants was assured, only the moderator and assistant had access to the data. Names and private information were not used in the transcripts and final report. The study was approved by Maastricht University’s Ethics Research Board of Psychology.

The topic list was constructed based on themes derived from available literature and in consultation with experts. The focus group topic list was pre-tested with colleagues and afterwards revised. All focus groups were semi-structured and the discussion proceeded in three parts: it started with an opening question in which participants introduced themselves and expressed whether or not they visited a CWC. The second part focused on participants’ vaccination decision-making process; questions were asked about the influence of social environment, role of culture and religion, role and assessment of received information, knowledge level concerning NIP-vaccinations, and possible practical barriers. In the third part, supplemental information was gathered about satisfaction of the participants with the NIP, if they would like to see some changes within the NIP, and their opinion about possible future vaccinations within the NIP.

### Analysis

The focus groups were audio taped and transcribed verbatim. Qualitative computer software MAXQDA (VERBI Software, Germany) version 10 was used to analyze the content of the focus group transcripts. To identify themes and sub-themes, thematic analysis was performed [14]. Separate analyses were performed for the Moroccan, Turkish, and mixed groups, and identified themes were compared between the groups. A coding frame was developed and transcripts were coded and analyzed by the moderator (HB). Initial codes were assigned to text fragments, and then were refined and arranged in themes and sub-themes. To enhance the reliability of data analysis, a sample of the transcripts was coded independently (IH). Afterwards, comparison of the codes took place and differences were discussed until consensus was reached. By using this method together with peer debriefing during the research process, researcher bias was reduced.

## Results

Four main themes were extracted from the focus group discussions. Some of the main themes were divided into sub-themes. The themes are summarized below with relevant quotes of participants. Results of Moroccan, Turkish, and participants with other ethnic backgrounds were described together, because the findings were mostly similar.

### Study participants

All participants were female. Most participants had one child (*n* = 13), 8 participants had two children, 5 participants had three children, four participants had four children, two participants had six children, and for one participant, the family size was unknown.

### Participation NIP and child welfare center

All participants confirmed that their children so far had received all NIP-vaccinations, corresponding to the Dutch NIP schedule.

#### Child welfare center visitation

Participants from all groups were familiar with the CWC for both health check-ups and receiving vaccinations for their child. Nearly all participants visited the same CWC in their neighborhood, and perceived CWC visitation as self-evident and important: *‘I visit with my child the CWC. We [the mothers] all have the same opinion, that it is just very important’* (Moroccan participant). Another Moroccan participant said: *‘Yes, it is obvious; it is just a normal thing to do [visiting the CWC]’.*

#### Accessibility

For almost all participants, the CWC was well accessible: *‘No, I have no problems in accessing the Child Welfare Centre’* (Turkish participant). A few participants mentioned that the CWC is located too far from their homes, and some participants mentioned that the distance negatively influenced the CWC visitation, but not when her child had to receive vaccinations: *‘I didn’t go the first time, I had an appointment, but I didn’t go. It [the CWC] was just too far away. After that I did go, because they [children] needed vaccinations, so I went there because of the vaccinations, otherwise I wouldn’t go there’* (Moroccan participant). Another participant said*: ‘I think the CWC is located too far, especially when the weather is bad and I need to walk because I do not have a car’* (Moroccan participant).

#### Satisfaction with CWC services

There were mixed findings about the satisfaction of the provision of NIP vaccinations, health check-ups and the approach of childhood vaccine providers (CVPs). Most participants were unsatisfied with the limited consultation time. Moroccan and Turkish participants indicated that they did not receive enough attention, and that there was not enough time to ask questions during CWC visitations*: ‘Here [at the CWC] everything goes quick, quick, quick’* (Moroccan participant)*.* Another Turkish participant said: *‘They never have a conversation with you; consults always take place automatically and very quick. Many parents regret that because they don’t receive enough attention.’* In the mixed groups, participants emphasized that they did not receive enough information, but they perceived the CWC as accessible for asking questions*: ‘They do not further explain what it means. That's really a shame, I think. They give the injections but they do not explain how and what. However, when I do have questions, I think it is possible to ask them’* (Participant mixed group). Two Turkish participants were not satisfied about the vaccination skills of the CVPs, but they indicated that this did not influence their decision to vaccinate*: ‘Some CWC nurses, they don’t inject well. They are so rough when they give the injection. I did not like that but I did not say anything about it’* (Turkish participant).

### Factors influencing parental decision making

Several factors influenced the decision of participants to vaccinate their child, such as their attitude towards the NIP, cultural and religious aspects, perceived social norm, negative experiences with vaccination and adverse events, knowledge level and understanding of the NIP, and practical issues.

#### Attitude towards vaccination

Almost all participants had a positive attitude towards childhood vaccination and mentioned that vaccination of their children is important because it benefits and protects their children’s health: *‘I did not really thought about whether to vaccinate or not, I thought it is just normal, you should be protected against diseases. I actually thought it was necessary. You hear it for years that children are being vaccinated and we have all been vaccinated ourselves. So it is just logical that they [the children] get vaccinated’* (Turkish participant). Most participants perceived vaccination as self-evident and some participants thought that participating in the NIP is mandatory: *‘It is so obvious that you think that it is obligatory’* (Moroccan participant). Turkish participants emphasised that it is important and logical to follow the advice of experts and CVPs*: ‘So very often you think as a parent; they are the experts, they know better than us’* (Turkish participant)*.* Another Turkish participant said: *‘If the CVP says it is good, then I assume that it is good. I have confidence in their advice’*.

#### Cultural aspects and religion

Most of the (Muslim) participants indicated that according to Islam, vaccination was considered as something beneficial: *‘Our faith tells us that we must protect our body well. That is our starting point’* (Turkish participant). Another participant said: ‘*We also get vaccinated in Morocco, it is just important to protect your children against diseases’* (Moroccan participant).

#### Perceived social norm

Most participants indicated that they had no conversations with neighbors, friends or family about vaccinations. *‘No, nobody talks about it. I think most mothers realize that it is very important and that it is something that has to be done for the health of their child’* (Moroccan participant). Although most participants did not discuss vaccination with their social environment, for some Turkish participants’ feelings of uncertainty about negative CWC experiences were a subject to discuss with friends or neighbors: ‘*I asked my friend how it went with vaccinating their child. She told me the same, that they gave the injection all of a sudden. So I thought: okay, it is normal’* (Turkish participant).

### Negative experiences with vaccination and adverse events

Some participants had experience of adverse reactions after vaccinating their child: *‘After my daughter was vaccinated she was sick for a week, she had 40° of fever and vomited. I thought for 8 days that my daughter was dying’* (Moroccan participant). Although this parent had a negative experience with vaccination, this did not influence her decision for future vaccinations: *‘No, I continued to have my daughter vaccinated’* (Moroccan participant). A participant from the mixed group became more afraid of vaccinations because her baby recently had febrile seizures after vaccination: *‘And now I am afraid because every time he gets a shot, he gets high fever. That is why I am afraid of the next shot: will he be okay this time?’* (Participant mixed group).

Some Turkish participants were sometimes worried about the vaccinations: *‘The first time, with your first child, you worry more; what is going to happen, how does he or she respond? I was worried’* (Turkish participant). Another Turkish participant said: *‘You do not get answers about the cause of the reaction. That fear stays in your mind’.* Nevertheless, most participants continued vaccinating their child*: ‘Yes, even if there are side effects, I will continue with vaccinating my child. Each drug has side effects, then you also do not quit, you also proceed’* (Turkish participant).

#### Transition and practical issues

None of the participants had problems with missing vaccinations because of transition to The Netherlands: *‘No, it was not a problem. I was instantly referred to the child welfare center when I came from Barcelona to the Netherlands’* (Moroccan participant).

Participants perceived no problems in receiving vaccination invitations or responding to calls. CWC appointments were clear and feasible to them: *‘Yes, everything was clear to me, I never had problems with that’* (Moroccan participant). Some participants had questions about how tocontinue the vaccination schedule when they went on holiday to their home country, but none of them missed vaccinations due to holiday abroad: *‘Then I called [the CWC] and they rescheduled my appointment’* (Turkish participant).

### Level of knowledge and understanding NIP

The majority of participants perceived their knowledge of vaccinations and the NIP as insufficient. Most participants know in general when the vaccines are given, but do not know against what infectious diseases the vaccines will protect: *‘You don’t know what these injections are for. You only hear the abbreviation [of the vaccine] when they are given, but not for what kind of diseases the injections are for’* (Moroccan participant*). ‘You have them [the children] vaccinated but you don’t know what kind of vaccinations they get’* (Turkish participant).

### Information

Several factors concerning NIP-information were extracted from the focus groups: evaluation of the received NIP information, perception of the language of the received information, information-seeking behavior, and information need of the participants.

#### Evaluation of received information

Almost all Moroccan participants evaluated the amount and content of information they received from CVPs as insufficient: *‘Because they don’t give you an explanation during vaccination…you just receive the jabs and you are finished. How many shots you get and for which diseases, that has actually never been told’* (Moroccan participant).

Among Turkish participants, experiences with receiving information varied. Turkish participants were more satisfied with the amount and content of information they received from CVPs and the Public Health Institute (PHI): *‘Yes, it was sufficient’* (Turkish participant). Some participants received information about vaccination when they visited the CWC: *‘Yes, when I went to the CWC they explained what can happen, or told me that I need to keep an eye on something. They told me that every time I visited the CWC’* (Turkish participant). Others said they did not receive information: *‘No, I did not get information. Normally in Turkey, they give some explanation before injecting, but in the Netherlands I did not get that’* (Turkish participant). In the mixed groups, most participants indicated that they received information by mail but they received little or no information from CVPs: *‘You do not really receive information about it. You only receive the information leaflet’* (Participant mixed group).

The information leaflet from the Public Health Institute (PHI) with information about the NIP was received and read by most Turkish participants: *‘Yes, I received it [the leaflet] together with the invitation letter’* (Turkish participant). Among Moroccan participants, the leaflet was less well known: *‘I never received it. I never received information about vaccinations’* (Moroccan participant). The leaflet was poorly read by Moroccan and Turkish participants: *‘No I never read it’* (Moroccan participant mixed group). Only some participants from the mixed group read it: *‘Yes I have read the brochure’* (Participant mixed group).

#### Perception of language received information

In both Moroccan and Turkish groups, the language of the education material was considered an obstacle for reading and understanding the content. Moroccan participants emphasized that there is a substantial group of Moroccans who do not understand the Dutch language, and therefore are not able to read the information leaflet in Dutch: *‘There are many people here in the district who can’t speak the Dutch language and are not able to read it. So, I think when you give a leaflet, it is important to give it in their own language too’* (Moroccan participant). Also in the Turkish group, a few participants could not read the information material due to language barrier: ‘*It is difficult for me; I do not understand the Dutch language’* (Turkish participant). This was the same for the non-Dutch participants in the mixed group: *‘I have problems with the Dutch language. I cannot read Dutch, I do not understand all the words’* (Non- Dutch participant mixed group). Some participants asked their husband or a friend to translate the information: *‘My husband reads it for me, explains to me what it means’* (Moroccan participant mixed group).

Most Turkish and Moroccan participants would like to receive information in respectively Turkish, Arabic and Berber language:*’Yes I want it in Turkish… because in that case, I know at least why my child receives that vaccination, otherwise I don’t know’* (Turkish participant). Participants indicated that provision of information in their own language would not influence their vaccination decision: *‘I don’t think so. I will get the vaccines, no matter what’* (Turkish participant).

#### Information seeking behavior

Most of the time, the participants used the CVPs and the Internet as a source to get information about vaccination. Some Turkish participants searched for additional information in their own language on the Internet, or asked questions at CVPs: *‘In advance I thought about what questions I would like to ask, what I wanted to know and then she [the CVP] explained it…because of her explanation I knew what to expect’* (Turkish participant).

Some Moroccan participants and participants from the mixed groups mentioned that they sometimes asked for more information about vaccination at CVPs: *‘Yes, I ask questions before my child gets the injection. I ask first, and then my child gets the vaccination’* (Participant mixed group). Sometimes (when possible) they talked with a doctor in their own language. Other Moroccan participants did not realize that they could ask for more information, or time constraints kept them from asking for information: *‘No, at that time, you don’t think about it’* (Moroccan participant).

Some participants searched for online information when there was a possible side effect: ‘*I will search for information on the Internet, if I know my child gets ill from the injections. When everything goes well, I will not search for information’* (Moroccan participant). In the mixed groups, the use of the Internet varied. Some participants indicated that they regularly used the Internet to find more information about vaccinations: *‘I read a lot in my own language. I search for information on the Internet’* (Participant mixed group). Most of them used the search engine Google. Other participants did not search online for vaccination information: *‘I never searched for information myself. I don’t know why…I just not really thought about it’* (Participant mixed group).

#### Information need

Most participants preferred more oral information from CVPs during CWC consultations: *‘That they explain where the vaccinations are for, that they give more face-to-face information during the consult, not that you just have to get your information from the leaflet. I prefer spoken information, because I will remember it better’* (Participant mixed group).

Participants would like to be more informed about the targeted infectious diseases and the prevalence of the diseases: *‘More information about the vaccinations themselves [….] more information about where the vaccinations are good for, and what they protect for’* (Moroccan participant).

Moroccan participants also desired more information about drawbacks of vaccination from the RIVM, because it would enable them to make a well-informed decision: *‘You hear things in the media but you don’t know whether that information is right. I would like to receive complete information from the RIVM with advantages and disadvantages. When we hear the pro’s and con’s from the RIVM about vaccination, we can make a better choice’* (Moroccan participant).

### Attitude towards future vaccinations

Participants had different opinions about possible future NIP vaccinations. Some participants were suspicious towards new vaccinations: *‘Vaccination against diphtheria or tetanus, that kind of diseases, is required for all your children, you simply choose for that. But for new vaccines, which are new to the market and are not thoroughly investigated, I have doubts about that’* (Turkish participant). In case of new vaccines, parents would like to receive information about reasons for introduction of the new vaccine, severity of the disease and the risk for their children to get the disease: *‘I would think about it and would like to know what kind of vaccine it is, against what kind of disease it will protect, and why the vaccine is introduced. In case of a new vaccine, I would not just vaccinate my child.’* (Turkish participant). Other participants were less critical towards possible new future NIP- vaccinations: *‘It does not matter to me, if it is necessary, then it is necessary. I assume that every vaccination, when added, is needed’* (Participant mixed group).

## Discussion

This study explored factors that influenced decision-making about childhood vaccination among parents with different ethnic backgrounds. Results show that the majority of parents made the decision to vaccinate their child(ren) based on a general positive attitude towards childhood vaccination, a high confidence in the advice of CVPs, and their religion-based positive beliefs about childhood vaccination. For some parents, there was a language barrier in understanding the provided NIP-information, the distance to the CWC was too far, and they indicated to have a need for more NIP- information.

Most parents in this study had a positive attitude towards childhood vaccination, and perceived vaccination as self-evident, which is also shown in a study of Streefland et al. [1]. Furthermore, Paulussen et al. [15] showed that most indigenous Dutch parents had a positive attitude towards childhood vaccination, and the decision for vaccination is also not preceded by extended reflection.

Cultural aspects and religion (e.g. Islam) seemed to have a positive influence on the decision of Turkish and Moroccan parents to vaccinate their child. According to the parents, Islam indicates that protecting the child’s health by means of vaccination is something beneficial. This is not in line with orthodox protestant religion in The Netherlands, which is shown to be highly related to vaccination refusal [4].

Some parents had a negative experience with vaccinating their children (adverse events), but this experience did not influence their decision to vaccinate. This is not in line with other research that showed that parents who had a negative experience with childhood vaccination were less likely to accept future vaccines [5, 16, 17].

CWC-visitation for vaccination is common among Moroccan and Turkish parents. It is shown that in the Netherlands 89 % of parents with different ethnic backgrounds and 91 % of indigenous Dutch parents visit the CWC [18]. Although most parents in our study visited the CWC, some parents indicated that they did not visit CWCs because the distance from their home was too far, which is in line with earlier research [19]. Especially when they had no access to a car and the weather was bad, they did not visit the CWC. Most parents indicated that they always visited the CWC when the child needed a vaccine, which was not the case for health check-ups. Although CWCs are well accessible in the Netherlands, the average distance to a Dutch CWC is 2.5 km [20], the access to the CWC seems to be a barrier for parents. More (quantitative) research is needed to get insight into how often parents do not visit the CWC because the CWC is located too far, and whether this influenced attending health check-ups and/or vaccination appointments.

Other practical barriers, like often changing their residence, children born abroad, and unfamiliarity with the health system [7] did not seem to play a role in the vaccination decision of the parents. Van der Wal et al. [21] indicated that vaccination coverage of children who were born abroad was not well registered and therefore vaccination coverage seemed lower. This barrier is not shown because this study focused at parents’ beliefs and personal barriers, therefore more research is needed to explore whether under-registration is an issue.

This study showed that the language of the information material was considered an obstacle in understanding the provided information, and parents would like to receive the education material in their native language (i.e., Turkish, Arabic or Berber). Language barriers were suggested in other studies to play a possible role in the vaccination decision-making process of parents with different ethnic backgrounds [6, 7]. In the Netherlands, there is a policy that the information from the government and public health institutes should be in Dutch, to stimulate citizens to learn the Dutch language [22]. However, we suggest providing NIP-information material in parents’ native language for parents who cannot read and understand the Dutch language. This might enable the parents to make better-informed decisions.

Moroccan and Turkish parents perceived their knowledge about vaccination as insufficient, and they have a need for more information about the NIP, which is in line with other research [23, 24]. Parents would specifically like to get more information about the diseases that the vaccinations protect for, and not only information about the advantages but also the possible disadvantages of vaccinating their children. They prefer to receive the information orally, which is in line with a study of Hak et al. [25] among Dutch parents. The preference of oral information is also reflected in the fact that the provided information leaflets were poorly read, also by parents who understand the Dutch language, a finding that is supported by Timmermans et al. [26].

Overall, parents had a lot of confidence in the advice of CVPs to vaccinate their children, which is also shown in other studies [1, 17]. Despite this, most parents were unsatisfied about the limited consultation time during CWC-visits. They felt that they did not receive enough attention from CVPs, and there was limited time to ask questions. Earlier research showed that CVPs indicated that there is limited time to discuss vaccination with parents [6]. CVPs should be aware of the oral information need of parents with different ethnic backgrounds and their role in parents’ decision-making process, and should therefore actively provide information towards parents about the NIP. Due to the limited time for CVPs to provide information that the parents need, it might be useful if CWCs organize meetings to provide (oral) information about the NIP in different languages.

Some limitations of this study need to be considered. By including Moroccan and Turkish parents, an important insight in influencing factors among the two biggest non-Dutch ethnic groups in The Netherlands is reached. Nevertheless, including parents of different ethnic backgrounds like Antillean or Surinamese participants, the third biggest non-Dutch ethnic group in the Netherlands, might also have been useful. Another limitation of this study might be selection bias, because all participants of the focus groups were women, attended regular meetings at welfare organizations and completely vaccinated their children according with the NIP. It might therefore be that the participants of the focus groups are somewhat more positive towards the NIP than other non-Dutch ethnic parents. Therefore, future research should be conducted to get insight in the factors that influenced parents whose children are not (completely) vaccinated. In addition, future research should also try to include more fathers in focus group studies to find out if there is a difference in vaccination decision making between non-Dutch ethnic mothers and fathers. While this qualitative study provides useful insight in acceptance of childhood vaccination and factors that influence decision-making about vaccination of parents with different ethnic backgrounds, quantitative confirmation of the findings is recommended among a large population of parents with different ethnic backgrounds.

## Conclusion

This study showed that parents with different ethnic backgrounds had a positive attitude to vaccinate their child within the NIP, and perceived vaccinating their child(ren) as self-evident. Parents perceive practical barriers like the distance to the CWC and not understanding the Dutch language. Furthermore, parents had a need for more NIP information. These findings suggest that information provision about the NIP towards parents with different ethnic backgrounds deserves extra attention. To fulfill the information need of the parents, more information about targeted diseases and complete information regarding benefits and drawbacks of the NIP should be provided. This should be provided not only by the PHIs, but also by CVPs, because parents with different ethnic backgrounds have a lot of confidence in CVPs, and have the preference to receive oral information. To fulfill parents’ information need, NIP information meetings can be organized at CWCs in different languages. Investigation of the utility of providing NIP information material in Turkish, Arabic and Berber language is also recommended. Providing information tailored to these parents’ needs is important to sustain vaccination participation, and can be of influence in accepting future vaccinations.
